# Fine-Scale Temporal Variation in Marine Extracellular Enzymes of Coastal Southern California

**DOI:** 10.3389/fmicb.2012.00301

**Published:** 2012-08-17

**Authors:** Steven D. Allison, Yi Chao, John D. Farrara, Stephen Hatosy, Adam C. Martiny

**Affiliations:** ^1^Department of Ecology and Evolutionary Biology, University of California-IrvineIrvine, CA, USA; ^2^Department of Earth System Science, University of California-IrvineIrvine, CA, USA; ^3^Remote Sensing Solutions, Inc.Pasadena, CA, USA; ^4^Joint Institute for Regional Earth System Science and Engineering, University of California-Los AngelesLos Angeles, CA, USA

**Keywords:** coastal ocean, extracellular enzyme, marine microbes, particles, phytoplankton, southern California, temporal variation, time series

## Abstract

Extracellular enzymes are functional components of marine microbial communities that contribute to nutrient remineralization by catalyzing the degradation of organic substrates. Particularly in coastal environments, the magnitude of variation in enzyme activities across timescales is not well characterized. Therefore, we established the MICRO time series at Newport Pier, California, to assess enzyme activities and other ocean parameters at high temporal resolution in a coastal environment. We hypothesized that enzyme activities would vary most on daily to weekly timescales, but would also show repeatable seasonal patterns. In addition, we expected that activities would correlate with nutrient and chlorophyll concentrations, and that most enzyme activity would be bound to particles. We found that 34–48% of the variation in enzyme activity occurred at timescales <30 days. About 28–56% of the variance in seawater nutrient concentrations, chlorophyll concentrations, and ocean currents also occurred on this timescale. Only the enzyme β-glucosidase showed evidence of a repeatable seasonal pattern, with elevated activities in the spring months that correlated with spring phytoplankton blooms in the Southern California Bight. Most enzyme activities were weakly but positively correlated with nutrient concentrations (*r* = 0.24–0.31) and upwelling (*r* = 0.29–0.35). For the enzymes β-glucosidase and leucine aminopeptidase, most activity was bound to particles. However, 81.2% of alkaline phosphatase and 42.8% of *N*-acetyl-glucosaminidase activity was freely dissolved. These results suggest that enzyme-producing bacterial communities and nutrient dynamics in coastal environments vary substantially on short timescales (<30 days). Furthermore, the enzymes that degrade carbohydrates and proteins likely depend on microbial communities attached to particles, whereas phosphorus release may occur throughout the water column.

## Introduction

Primary production in the ocean is influenced by the regeneration of nutrients through the microbial loop (Azam, 1998). Phytoplankton convert inorganic carbon (C), nitrogen (N), and phosphorus (P) into organic forms that are efficiently recycled back into inorganic molecules primarily through the activity of heterotrophic bacteria (Azam et al., 1992). A large fraction of the biomolecules synthesized by marine organisms are polymeric or too large to be transported across the cytoplasmic membrane (Arnosti, 2004). Therefore a critical component of the microbial loop is the extracellular hydrolysis of organic molecules by hydrolytic enzymes (Turley, 1994; Hoppe et al., 2002; Arnosti, 2011).

An extracellular enzyme is defined here as any enzyme that is located outside the cell membrane, either freely dissolved in the water column, bound to the cell surface, or localized in the periplasmic space. Periplasmic and bound enzymes may enable the cell to preferentially access hydrolysis products, but require the substrate to penetrate the cell wall or be physically located near the cell. Free extracellular enzymes may originate from secretion into the water column or release during cell lysis (Chróst, 1991). These enzymes can access distant substrates, but hydrolysis products may not return to the enzyme producer. Previous studies have shown that the amount of activity in free versus particulate water fractions varies widely (Bochdansky et al., 1995; Davey et al., 2001; Obayashi and Suzuki, 2008).

Extracellular enzymes are produced by a range of marine organisms. Phytoplankton and heterotrophic bacteria are known to produce alkaline phosphatase to acquire P from organic sources, particularly when inorganic phosphate is low in concentration (Hoppe, 2003). Other hydrolytic enzymes such as chitinases and peptidases involved in organic N cycling and glucosidases involved in carbohydrate degradation are more likely produced by bacterial heterotrophs (Garsoux et al., 2004; Dang et al., 2009; Zhou et al., 2009) or zooplankton (Oosterhuis et al., 2000; Vrba et al., 2004). However, the nutrients released by extracellular enzyme activity or the subsequent turnover and lysis of enzyme-producing microbes can potentially be accessed by all members of the microbial community (Hoppe et al., 2002).

Although extracellular enzymes are essential components of marine biogeochemical cycles, relatively little is known about the factors contributing to spatial and temporal variability in marine enzyme activities. There is evidence from lakes (Middelboe et al., 1995) and seawater mesocosms (Riemann and Steward, 2000) that phytoplankton blooms increase the availability of organic substrates targeted by extracellular enzymes and exploited by heterotrophic microbes. Whether there is consistent temporal coherence between phytoplankton blooms and enzyme activities in marine environments is unclear. Enzyme production could also be induced by low nutrient conditions resulting from phytoplankton uptake or the advection of nutrient-poor surface waters (Chróst, 1991). Enzyme activities could also depend on the dynamics of specific microbial taxa that possess genes for enzyme production (Martinez et al., 1996). Metagenomic and metatranscriptomic analyses from the English Channel have shown that the composition and functional potential of marine microbial communities varies on diel and seasonal timescales (Gilbert et al., 2010, 2012). Predictable seasonal patterns in microbial community composition have also been found in the Southern California Bight (Fuhrman et al., 2006), suggesting that there could be similar patterns in extracellular enzyme activity.

Our objective was to assess the timescales of variation in marine extracellular enzyme activities and ask whether this variation is correlated with other biophysical parameters in a coastal environment. Few studies have examined marine enzymes or microbial communities on timescales shorter than 1 month (Karner and Rassoulzadegan, 1995), yet there are many biophysical processes that operate on daily to weekly timescales. In particular phytoplankton blooms, winds, upwelling, and rainfall events can all vary on short timescales and could affect microbial processes in coastal environments (Santoro et al., 2010; Gilbert et al., 2012). These dynamics could lend insight into the mechanisms that control primary production and the timing of harmful algal blooms that occur in many coastal environments, including the Southern California Bight where our study was conducted (Schnetzer et al., 2007).

To examine these patterns and mechanisms, we established the MICRO (Microbes in the Coastal Region of Orange County) time series to examine dynamics in marine extracellular enzymes, chlorophyll concentrations, nutrient concentrations, and physical ocean conditions at high temporal resolution in a coastal environment. We hypothesized that extracellular enzyme activities would vary mainly at short timescales (<30 days) and would be positively correlated with nutrient and chlorophyll concentrations. We also predicted that there would be a repeatable seasonal pattern in enzyme activities. Finally, we hypothesized that enzyme activities would be localized in particulate fractions (cell-bound or particle-bound) because hydrolysis on particles or cell surfaces should allow direct coupling between enzyme production and nutrient uptake that would benefit the enzyme producer (Smith et al., 1992).

## Materials and Methods

### Sampling

Seawater was sampled up to three times per week from Newport Pier, Newport Beach, CA, USA (33.607°N, 117.930°W), between 08:00 and 09:00 local time from January 1, 2009, through January 1, 2012. The sampling site is ∼100 m from shoreline, and the seafloor is ∼25 m below the surface at the site. On each date, two independent surface water samples (depth <0.5 m) were collected in a clean 8 L bucket pre-rinsed with seawater. Each sample was divided into subsamples that were transported to UC Irvine at 15–20°C for further analysis within 30 min. One subsample was placed in an acid-washed 1 L polypropylene bottle and passed through 2.7 μm GF/D and 0.2 μm polyethersulfone filters in the laboratory. A 15-mL subsample of the filtrate was collected in an acid-washed 20 mL scintillation vial and frozen (−20°C) for nutrient analysis. A second set of three subsamples was taken at the pier and placed in pre-rinsed 20 mL scintillation vials for enzyme analyses. One enzyme subsample contained unfiltered seawater. The second (<2.7 μm) and third (<0.2 μm) subsamples contained the filtrate from seawater pushed by hand through 2.7 μm GF/D or 0.2 μm polyethersulfone syringe filters, respectively.

### Enzyme assays

Assays of potential activity of four extracellular enzymes (Table 1) were conducted on bulk seawater and the two filtered water fractions using model substrates tagged with fluorophores (Hoppe, 1983). The activities we report should be considered potential values because they were measured under high substrate concentrations and are more closely related to enzyme concentration than *in situ* activity (Wallenstein and Weintraub, 2008). Assays were conducted in black 96-well microplates (Greiner Bio-One) and run at 20°C for 1.5 h, a time period over which preliminary experiments showed the increase in fluorescence was generally linear.

**Table 1:** **Enzymes, substrates, and assay conditions**.

Enzyme	Enzyme function	Substrate proxy	Substrate concentration
Alkaline phosphatase (AP)	Hydrolyzes phosphate monoesters	4-MUB-phosphate (Sigma-Aldrich)	200 μmol L^−1^
β-glucosidase (BG)	Releases glucose from polysaccharides	4-MUB-β-d-glucopyranoside (Sigma-Aldrich)	40 μmol L^−1^
Leucine aminopeptidase (LAP)	Hydrolyzes polypeptides	l-leucine-AMC (Fisher Scientific)	80 μmol L^−1^
*N*-acetyl-glucosaminidase (NAG)	Releases *N*-acetyl-glucosamine from chitin	4-MUB-*N*-acetyl-β-d-glucosaminide (Sigma-Aldrich)	80 μmol L^−1^

*MUB, methylumbelliferyl; AMC, 7-amido-4-methylcoumarin*.

For each water fraction, we added 50 μL substrate solution to a 200-μL sample to initiate the reaction. Sample blanks (200 μL sample + 50 μL DI water) were included to account for the background fluorescence of each water fraction. Substrate blanks (200 μL filtered, autoclaved seawater + 50 μL substrate solution) were included to account for the autohydrolysis of the substrate during the assay incubation. Fifty microliters of standard solution (10 μM 4-methyl-umbelliferone or 10 μM 7-amino-4-methylcoumarin, Sigma-Aldrich) was added to 200 μL sample to allow conversion of fluorescence into product concentration and to account for quenching of fluorescence by the seawater sample. Microplates were tapped gently to mix solutions and read at 360 nm excitation/460 nm emission (BioTek Synergy 4 microplate reader) at time zero, 0.5, 1.0, and 1.5 h. The concentration of reaction product [P] present in the wells was calculated as:

(1) $$\left[\text{P}\right]=\frac{F_{\text{Assay}}-F_{\text{Substrate blank}}}{\frac{F_{\text{Standard}}-F_{\text{Sample blank}}}{C_{\text{Standard}}\times V_{\text{Standard}}}\times V_{\text{Sample}}}$$

where *F* is fluorescence, *C* is concentration, and *V* is volume. Potential enzyme activity (hereafter “activity”) in nmol L^−1^ h^−1^ was then determined as the slope of a regression of [P] versus incubation time. The fluorescence values used for standards and blanks in Eq. 1 represent the mean value of six of eight replicate wells; the two-wells with fluorescence values most distant from the eight-well mean were removed. Similarly, eight replicate sample wells were used to generate eight regression slopes for each sample, and the two most deviant slopes were removed prior to calculating the mean enzyme activity. Standard fluorescence values on each sampling day were normalized to a 14-day moving average (with outliers removed) to minimize any variation introduced during the daily preparation of standards. Together, these data processing steps reduce assay variation due to pipetting errors.

Enzyme assays on seawater passing through 2.7 and 0.2 μm filters were used to calculate the activity in four water fractions. If the measured activity passing through the 2.7-μm filter exceeded the activity in unfiltered seawater (for instance due to cell lysis during filtration), then the activity in the <2.7-μm subsample was set equal to the unfiltered value. Similarly, the activity passing through the 0.2-μm filter was not permitted to exceed the activity passing through the 2.7-μm filter (or the activity in unfiltered seawater). We define the “free” fraction to include enzymes in the <0.2-μm subsample that are likely freely dissolved in seawater. The “small” fraction includes enzymes bound to particles between 0.2 and 2.7 μm in size; this fraction was calculated by subtracting the free fraction from the activity in the <2.7-μm subsample. The “large” fraction includes enzymes bound to particles >2.7 μm and was calculated by subtracting the activity in the <2.7-μm subsample from the activity in unfiltered seawater. The “particulate” fraction includes enzymes bound to particles >0.2 μm and was calculated by subtracting the free fraction from unfiltered seawater. The activity *A* in each fraction *j* is reported as a percentage of the unfiltered fraction *A*_U_, averaged over *n* dates:

(2) $$\frac{\overset{n}{\underset{i=1}{\sum }}{\left(\frac{A_{j}}{A_{\text{U}}}\times 100\right)}_{i}}{n}$$

### Pier-based nutrient concentrations, chlorophyll, and physical conditions

Frozen samples were shipped to the Marine Science Institute, Santa Barbara, CA, USA, for analysis of ${\text{NH}}_{\text{4}}^{+}{\text{, NO}}_{\text{2}}^{-}+{\text{NO}}_{\text{3}}^{-}\;\left(\text{N}{\text{O}}_{\text{x}}\right),{\text{ PO}}_{\text{4}}^{\text{3}-},\;\;\text{and}\;\;{\text{SiO}}_{\text{4}}^{\text{4}-}.$ Nutrients were analyzed by flow injection with detection limits of 0.1, 0.2, 0.1, and 1.0 μmol L^−1^, respectively. Samples with concentrations below detection were set to one-half the detection limit.

We obtained temperature, salinity, and chlorophyll data for the duration of the time series from the Southern California Coastal Ocean Observing System website^1^. Temperature and salinity data were generated by an automated sensor located on Newport Pier ∼3 m below the surface. Measurements taken at 4 min intervals were used to calculate daily averages. Chlorophyll data were derived from surface samples taken weekly from Newport Pier and analyzed by the University of Southern California as part of a monitoring program for harmful algal blooms.

### Offshore chlorophyll, sea surface temperature, and currents

Enzyme activities in seawater sampled from Newport Pier may be influenced by environmental drivers across a range of spatial scales. To assess these drivers beyond the local scale, we analyzed satellite data and ocean model outputs for a ∼3600-km^2^ offshore region surrounding Newport Pier. Level-2 ocean color and 11 μ sea surface temperature (SST) .hdf files from MODIS *Aqua* were downloaded from the NASA Ocean Color website^2^. We selected a region from 32°N to 34°N and 117°W to 119°W from January 1, 2009 to January 1, 2012. Level-2 files were processed to Level-3 files at 1 km resolution using custom Unix shell scripts and the *l2bin* function of the SeaDAS software package^3^. Level-3 files were further binned using the *l3bin* function in SeaDAS to calculate daily 3-day moving average SST or ocean color values at 1 km resolution. These daily composite data were output along with latitude and longitude for each pixel using custom IDL scripts with the *out_ascii* function. Using an ocean mask, a subset of pixels from these outputs was selected within a roughly 45 km × 80 km region including the San Pedro basin to Catalina Island and the coastal ocean south to San Clemente, CA, USA. This region was intended to represent the offshore environment that could influence enzyme activities at Newport Pier on daily to weekly timescales. All non-missing SST or chlorophyll values within this region were averaged to generate a daily time series of values for the study period.

Physical ocean parameters were calculated for the same region and time period using historical predictions from a ROMS model (Chao et al., 2009). The vector fields from ROMS were used to calculate daily mean alongshore and across-shore currents for the surface 50 m. Positive alongshore currents correspond to movement from Mexico toward Pt. Conception; positive across-shore currents correspond to movement toward the shoreline. ROMS reliability was assessed by comparing SST predictions from ROMS to the SST observations from MODIS. Temperature predictions from the ROMS model for the Newport Pier area were highly correlated with MODIS satellite observations (*r* = 0.89, *P* < 0.001) and automated temperature measurements at the pier (*r* = 0.87, *P* < 0.001), suggesting that ROMS predictions of physical conditions are valid at the MICRO site. We also downloaded the daily Bakun upwelling index for 33°N, 119°W from the NOAA Pacific Fisheries Environmental Laboratory^4^ as an indicator of upwelling strength in the Southern California Bight (Bakun, 1973). These values are derived from synoptic (6-hourly) sea level pressure gridded fields and are reported in units of m^3^ s^−1^ 100 m^−1^ coastline. All physical ocean parameters were averaged using a 3-day moving window.

### Statistical analyses

Data were converted to daily timeseries using the *timeseries* R package with linear interpolation where necessary (R Development Core Team, 2011). We used the autocorrelation function at lags of 0–365 days to determine if there were seasonal patterns in our study variables. Data variance was partitioned into different timescales with the *spectrum* function in R, which uses fast Fourier transform to calculate spectral density as a function of frequency. Data were detrended, demeaned, and analyzed with 3 and 5 days modified Daniell smoothers. Variance was assigned to sub-weekly, weekly monthly, monthly seasonal, seasonal-annual, and interannual timescales based on periods of 2–7, 7–30, 30–90, 90–365, and >365 days, respectively. We restricted our time series analyses of enzyme activities to the unfiltered fraction since this fraction contains all of the other fractions.

Relationships between variables were assessed using the cross-correlation function in R. This function calculates the correlation between two timeseries as a function of time lag. We checked all lags between 0 and 60 days and extracted the highest correlation coefficient over this interval and its associated lag. We tested the statistical significance of each correlation by constructing a 95% confidence interval based on *N*_e_ effective degrees of freedom (Dawdy and Matalas, 1964):

(3) $$N_{\text{e}}=\frac{N\left(1-r_{1x}\times r_{1y}\right)}{1+r_{1x}\times r_{1y}}$$

where *N* is the number of observations and *r*_1*x*_ and *r*_1*y*_ are the lag-1 autocorrelations for the *x* and *y* time series. The 95% confidence interval is then defined as:

(4) $$\text{C}{\text{I}}_{95}=0\pm \frac{1.96}{\sqrt{N_{\text{e}}-2}}$$

Correlation coefficients lying outside this interval were considered statistically significant at *P* < 0.05 unless otherwise noted. For relationships involving pier-based chlorophyll and temperature measurements, we also calculated Pearson correlation coefficients at zero lag without data interpolation. However, we determined statistical significance based on effective degrees of freedom from the interpolated time series if *N*_e_ was less than the degrees of freedom for the Pearson correlation (i.e., if the data were highly autocorrelated). We also tested the combined contributions of multiple environmental drivers to variation in enzyme activities at zero time lag using stepwise multiple regression (*MASS* package in R). We conducted separate regressions with MODIS chlorophyll and pier-based chlorophyll since the latter was sampled only once per week. Enzyme activities, chlorophyll concentrations, and nutrient concentrations were log-transformed prior to regression analyses.

## Results

### Distribution of enzyme activities

To characterize temporal patterns in marine extracellular enzymes, we measured enzyme activities three times per week for 3 years. We observed that the distribution of activity across water fractions varied according to enzyme (Table 2). A large proportion of AP activity was found in the free fraction, with most of the remaining activity in the large fraction. We also found a 42.8 ± 0.8% of NAG activity in the free fraction, with most of the remaining activity in the large fraction, suggesting that this enzyme is either freely dissolved in seawater or bound to large particles. In contrast to AP and NAG, most BG and LAP activity was bound to cells or particles. The large fraction contained the most BG (59.9 ± 0.9%), followed by the small fraction with 26.7 ± 0.7%, and only 13.4 ± 0.6% in the free fraction. LAP activity was distributed similarly.

**Table 2:** **Mean (±SE) percentage enzyme activity in seawater fractions as a proportion of the enzyme activity in unfiltered seawater**.

	Free		Small		Large		Particulate	
	Mean	±SE	Mean	±SE	Mean	±SE	Mean	±SE
AP	81.2	0.9	4.1	0.4	14.7	0.8	18.8	0.9
BG	13.4	0.6	26.7	0.7	59.9	0.9	86.6	0.6
LAP	13.3	0.6	18.4	0.6	68.3	0.8	86.7	0.6
NAG	42.8	0.8	5.1	0.5	52.1	0.9	57.2	0.8

*Particulate = 100 − free fraction. AP, alkaline phosphatase; BG, β-glucosidase; NAG, *N*-acetyl-glucosaminidase; LAP, leucine aminopeptidase*.

### Range and timescales of variation

**Figure 1:**
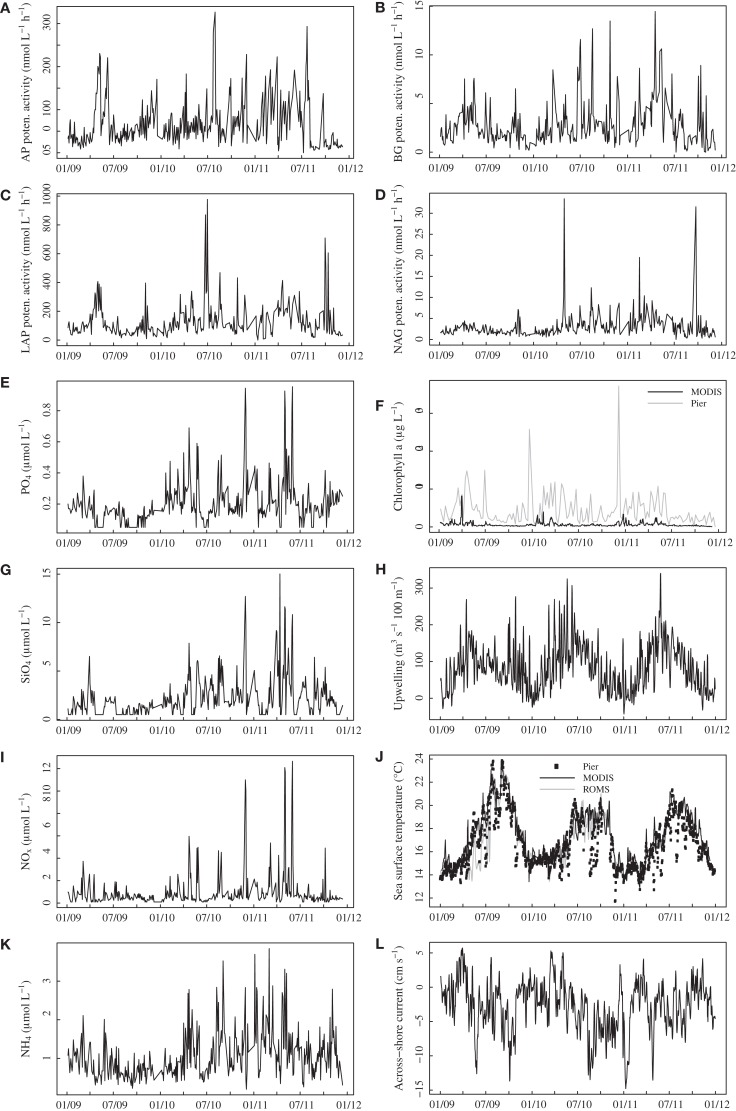
**Time series plots for bulk seawater extracellular enzyme activities (A–D) and seawater nutrient concentrations (E,G,I,K) in surface water at Newport Pier**. Chlorophyll a data **(F)** are derived from MODIS satellite data for a ∼3600-km^2^ region surrounding Newport Pier (black line) or from surface water samples taken weekly at the pier (gray line). Sea surface temperatures **(J)** are from an automated sensor at Newport Pier (squares), MODIS satellite data from the 3600-km^2^ region (black line), and ROMS model output from the 3600-km^2^ region. The Bakun upwelling index **(H)** is shown for the Southern California Bight at 33°N, 119°W. Across shore **(L)** currents were derived from ROMS model outputs (surface 50 m) for the 3600-km^2^ region. AP, alkaline phosphatase; BG, β-glucosidase; LAP, leucine aminopeptidase; NAG, *N*-acetyl-glucosaminidase.

All enzyme activities varied over several orders of magnitude throughout the time series, with much lower variation among replicate samples taken on the same day (mean coefficient of variation among replicates = 0.13, 0.13, 0.12, 0.15 for AP, BG, LAP, NAG, respectively). AP activities reached levels exceeding 200 nmol L^−1^ h^−1^ on several dates (Figure 1A), and BG activity showed a similar pattern of variation, exceeding 10 nmol L^−1^ h^−1^ on several days during summer-fall 2010 and spring 2011 (Figure 1B). LAP activities generally ranged from 50 to 300 nmol L^−1^ h^−1^ but exceeded 600 nmol L^−1^ h^−1^ during summer 2010 and fall 2011 (Figure 1C). NAG activities in the range of 2–5 nmol L^−1^ h^−1^ were punctuated by spikes reaching above 30 nmol L^−1^ h^−1^ in spring 2010 and fall 2011 (Figure 1D), with generally less variable activities in 2009.

Nutrient and chlorophyll concentrations also varied widely over the study period. PO_4_, SiO_4_, NO_x_, and NH_4_ concentrations ranged from below detection to >0.9, 15, >10, and >3.5 μmol L^−1^, respectively (Figure 1). As with NAG activities, most nutrient levels were less variable in 2009 than in 2010–2011. Average offshore chlorophyll concentrations based on MODIS were highest during spring and exceeded 8 μg L^−1^ in 2009 (Figure 1F). Pier-based measurements of chlorophyll were also elevated during the spring, with two large blooms (>25 μg L^−1^) during the winters of 2010 and 2011. The two chlorophyll measures were positively related, but the correlation coefficient was only 0.31 (*P* < 0.001), and the pier chlorophyll levels were much higher than the satellite-derived values. SSTs derived from MODIS ranged from 13 to 24°C, with the warmest temperatures in 2009 (Figure 1J). Although these values were highly correlated with temperatures from the automated pier sensor (*r* = 0.87, *P* < 0.001), the pier measurements showed rapid cooling events that were not captured by the satellite data. Ocean currents from ROMS ranged from −40 to 20 cm s^−1^ alongshore (not shown) and −15 and 5 cm s^−1^ across-shore (Figure 1L). The strongest upwelling generally occurred in the spring and early summer, with an additional period of upwelling in the fall of 2009 (Figure 1H).

**Figure 2:**
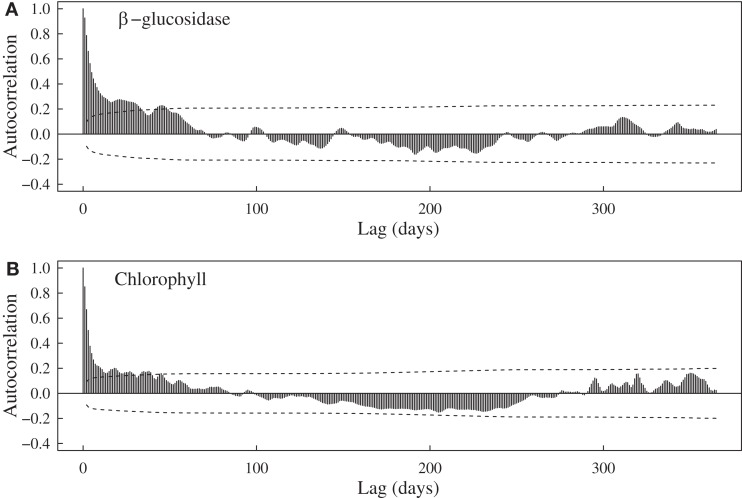
**Autocorrelation functions for (A) bulk seawater β-glucosidase activities at Newport Pier and (B) chlorophyll a concentrations derived from MODIS satellite data for a ∼3600-km^2^ region surrounding Newport Pier**. Dashed lines represent the 95% confidence interval.

**Figure 3:**
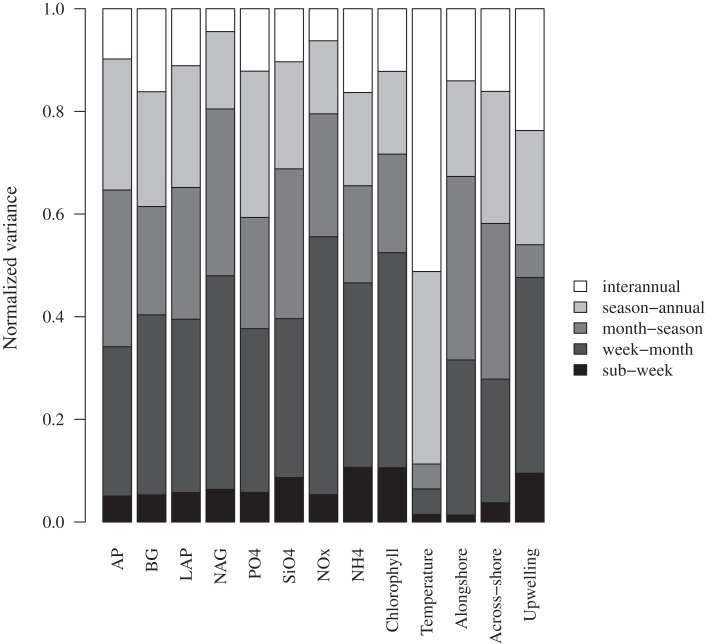
**Variance at different timescales for bulk seawater extracellular enzyme activities, seawater nutrient concentrations, chlorophyll a concentrations, and sea surface temperature**. The total variance for each variable was normalized to 1.0 to facilitate comparison. AP, alkaline phosphatase; BG, β-glucosidase; NAG, *N*-acetyl-glucosaminidase; LAP, leucine aminopeptidase. Alongshore and across-shore refer to surface current directions.

Aside from expected seasonal patterns in SST and upwelling, autocorrelations at seasonal lags were weak for most variables. However, there was evidence for a repeatable seasonal pattern in BG, with peak activities during late spring-early summer (Figures 1B and 2A). MODIS chlorophyll showed a similar seasonal pattern, likely due to spring phytoplankton blooms (Figures 1F and 2B). For all variables except SST, 28–56% of the variance in mean daily values occurred at timescales <30 days (Figure 3). For unfiltered enzyme activities, the fraction of variance at <30 day timescales ranged from 34% for AP to 48% for NAG. The <30-day fraction of variance was similar for nutrients, which ranged from 48% for ${\text{PO}}_{\text{4}}$ to 56% for NO_x_. Fifty-two percent of the variation in chlorophyll levels also occurred at timescales less than 30 days, whereas 94% of the variation in SST occurred at seasonal to interannual timescales.

### Relationships among variables

To identify potential mechanisms driving enzyme variation, we tested for correlations between enzyme activities and environmental parameters. We found that enzymes were weakly correlated with most other parameters, although there were some stronger relationships between enzymes (Table 3). AP activity correlated positively with BG (*r* = 0.41) and LAP (*r* = 0.39) after a 30-day lag. BG activity was also positively correlated with LAP (*r* = 0.76) and NAG (*r* = 0.34) at zero lag. Likewise, NAG and LAP were positively correlated (*r* = 0.38) at zero lag. Most enzymes showed weak positive correlations with nutrients (*r* = 0.24–0.31) and upwelling (*r* = 0.29–0.35), and there were positive or negative lags associated with most of these correlations. All enzymes except NAG were weakly positively correlated with MODIS chlorophyll and lagged it by 31–45 days. Zero lag correlations with pier-based chlorophyll levels were higher for BG (*r* = 0.39, *P* < 0.001), LAP (*r* = 0.48, *P* < 0.001), and NAG (*r* = 0.31, *P* < 0.01), although these relationships were based on a smaller subset of ∼95 dates. All enzymes except NAG were weakly negatively correlated with across-shore currents and led the currents by 33 to >60 days. BG and LAP were negatively correlated with MODIS SST and lagged it by ∼60 days. There were no negative relationships between enzyme activities and salinity, suggesting that enzyme dynamics were not driven by local precipitation inputs.

**Table 3:** **Significant (*P* < 0.05) cross-correlation coefficients and lags for bulk seawater enzyme activities, nutrient concentrations, MODIS chlorophyll (chl), and sea surface temperatures (SST), alongshore or across-shore ocean currents, and the Bakun upwelling index**.

	BG	LAP	NAG	PO_4_	SiO_4_	NO_x_	NH_4_	chl	SST	Along	Across	Upwelling
AP	0.41 (−30)	0.39 (−30)	NS	0.27 (−60)	0.31 (−60)	0.25 (−60)	NS	0.21 (−38)	NS	NS	−0.26 (33)	0.29 (−4)
BG	–	0.76 (0)	0.34 (0)	NS	0.31 (2)	0.24 (5)	0.26 (15)	0.22 (−45)	−0.35 (−60)	NS	−0.26 (59)	0.33 (15)
LAP	–	–	0.38 (0)	NS	0.31 (−26)	NS	0.23 (34)	0.23 (−31)	−0.27 (−58)	NS	−0.25 (60)	0.35 (−6)
NAG	–	–	–	NS	0.24 (26)	NS	0.29 (−7)	NS	NS	NS	NS	NS
PO_4_	–	–	–	–	0.66 (0)	0.83 (0)	0.52 (0)	0.24 (2)	−0.38 (0)	NS	NS	NS
SiO_4_	–	–	–	–	–	0.69 (0)	0.37 (0)	0.25 (2)	NS	NS	NS	0.24 (28)
NO_x_	–	–	–	–	–	–	0.43 (0)	0.27 (2)	NS	NS	NS	0.20 (27)
NH_4_	–	–	–	–	–	–	–	NS	−0.24 (−40)	NS	NS	NS
chl	–	–	–	–	–	–	–	–	−0.36 (−40)	NS	0.20 (1)	−0.25 (−58)
SST	–	–	–	–	–	–	–	–	–	NS	−0.29 (5)	0.49 (−59)
Along	–	–	–	–	–	–	–	–	–	–	0.52 (4)	NS
Across	–	–	–	–	–	–	–	–	–	–	–	NS

*The variable in the column lags the variable in the row by the number of days in parentheses. AP, alkaline phosphatase; BG, β-glucosidase; NAG, *N*-acetyl-glucosaminidase; LAP, leucine aminopeptidase*.

Multiple regression analyses showed that all environmental drivers together explained less than 20% of the variation in enzyme activities when MODIS-derived chlorophyll data were included (Table 4). Since many of the drivers correlate with one another (Table 3), combing them in a multiple regression explains relatively little enzyme variation. However, multiple regressions that included pier-based chlorophyll data were able to explain a larger fraction (21–51%) of the variation in enzyme activities.

**Table 4:** **Adjusted multiple coefficients of determination (*R*^2^), degrees of freedom (*d.f*.), and variables retained after stepwise selection in multiple regression analyses (abbreviations as in Table 3)**.

Enzyme		*R*^2^	*d.f*.	Variables retained in model
AP	Model 1	0.06	282	SST, PO_4_, NO_x_, upwelling
	Model 2	0.21	68	Pier chl, PO_4_, SiO_4_, NO_x_, across
BG	Model 1	0.14	282	MODIS chl, PO_4_, SiO_4_, NO_x_, NH_4_, upwelling
	Model 2	0.33	69	Pier chl, NH_4_, across, upwelling
LAP	Model 1	0.19	276	MODIS chl, PO_4_, SiO_4_, NO_x_, NH_4_, across, upwelling
	Model 2	0.51	71	Pier chl, NH_4_, upwelling
NAG	Model 1	0.15	283	MODIS chl, SiO_4_, NH_4_, upwelling
	Model 2	0.44	70	Pier chl, NH_4_, across

*Model 1 includes daily MODIS-derived chlorophyll values whereas Model 2 includes weekly pier-sampled chlorophyll values*.

Nutrient concentrations were positively correlated with one another at zero lag, with correlation coefficients ranging from 0.37 to 0.83 (Table 3). Correlations between nutrients and physical ocean parameters were generally weak, although PO_4_ and NH_4_ were negatively correlated with MODIS SST (*r* = −0.38 and −0.24, respectively), and SiO_4_ and NO_x_ were positively correlated with upwelling (*r* = 0.24 and 0.20, respectively). MODIS chlorophyll was weakly positively correlated with across-shore current and all nutrients except NH_4_, but negatively correlated with upwelling (*r* = −0.25) and SST (*r* = −0.36). Most of these correlations occurred with positive or negative lags, although we generally observed similar correlations at zero lag; only correlations with upwelling were not evident at zero lag.

## Discussion

Consistent with our initial hypothesis, we found that much of the variation in enzyme activities occurred at timescales shorter than 1 month. We also found that nutrient and chlorophyll concentrations as well as surface currents and upwelling showed similar patterns of short-term variation. Only SST varied mainly at seasonal and interannual timescales. These results indicate that the factors controlling enzyme activity, nutrients, and chlorophyll in this coastal environment are dynamic on short timescales, and monthly sampling of these parameters would miss nearly half of the variation we observed.

Our results contrast with longer time series that have examined microbial community composition. In particular, the SPOTS time series is also located in the Southern California Bight, and studies there have shown predictable seasonal changes in microbial community composition (Fuhrman et al., 2006). Microbial diversity and community structure in the English Channel were also related to seasonality as indicated by day length (Gilbert et al., 2012). In our time series, the seasonal component of variation was relatively small, and most of our parameters did not cycle in a repeatable manner across seasons. This difference could be due to variation in environmental drivers or different responses of microbial community composition versus enzyme activity. SPOTS is located ∼40 km offshore and may therefore be more strongly influenced by large-scale hydrographic factors such as upwelling and the El Niño-Southern Oscillation (Fuhrman et al., 2006). Alternatively, enzyme activities are a functional component of the microbial community that may show different patterns of temporal variation compared to overall composition. Consistent with this idea, microbial gene expression was more variable on diel than seasonal timescales in the English Channel, whereas microbial metagenome composition varied most strongly on seasonal timescales (Gilbert et al., 2010).

Our study is unique in its focus on multi-year dynamics of marine extracellular enzymes at high temporal resolution. Other studies have examined variation in extracellular enzyme activities across oceanographic provinces (Christian and Karl, 1995), latitude (Arnosti et al., 2011), and depth (Davey et al., 2001), but these provide little information on temporal variation. At a coastal site in the Mediterranean, glucosidase and LAP activities varied by a factor of 2–3 within a given day, but there were no apparent seasonal patterns in enzyme activity, and low sample sizes precluded the partitioning of variance across timescales (Karner and Rassoulzadegan, 1995). We observed a similar amount of intra-day variation in enzyme activities measured every 30 min over a 6-h period on March 12, 2010 (Allison, unpublished), but the range of activity (max–min) on that day was less than 15% of the range observed across our 3-year time series for all enzymes. Duplicate samples taken at roughly the same time at our study site also showed low variability with coefficients of variation <0.15. However, metatranscriptomic analyses from the English Channel show that gene expression by marine bacteria and archaea can vary strongly from day to night (Gilbert et al., 2010). Although we identified high variance in enzyme activities on daily to weekly timescales at our study site, additional analyses should be conducted to assess the amount variation occurring at timescales shorter than 1 day.

We observed a pattern of lower variation in NAG activity and nutrient concentrations during 2009 compared to 2010–2011. Although these patterns could have been due to unknown differences in our methodology over time, NAG activities and nutrients were sampled and analyzed by largely independent techniques. Rather, we speculate that the change in variation may have been related to ocean physical conditions. SSTs were higher in 2009, which may have led to greater stratification, reduced upwelling at our site, and lower nutrient and enzyme levels. The pier temperature data (Figure 1J) also show a higher frequency of cold water incursions in 2010–2011.

Relationships with oceanographic drivers provide some information on the possible mechanisms controlling marine enzyme activities. Although most of these relationships were weak, we did observe positive correlations between enzymes and chlorophyll concentrations. AP, BG, and LAP were significantly correlated with MODIS chlorophyll after lags of 30–45 days. Furthermore, BG activities showed some evidence of a seasonal cycle (Figure 2) that mirrored MODIS chlorophyll. Consistent with an earlier study in this region (Santoro et al., 2010), chlorophyll levels were elevated during spring and low in the fall (Figure 1F). Using pier-based data instead of MODIS data revealed even stronger relationships between enzymes and chlorophyll. This pattern is not surprising because there are often shifts in phytoplankton composition and sharp increases in chlorophyll levels close to shore in this region. Internal tides, winds, and currents can interact with the nutricline to increase chlorophyll concentrations nearly 10-fold over distances of ∼10 km (Lucas et al., 2011). Thus heterotrophic microbes at our coastal site may be responding to biomass and C compounds generated during phytoplankton blooms (Middelboe et al., 1995).

Variation in enzyme activity at Newport Pier is probably also affected by local processes such as wind patterns, waves, and runoff or groundwater discharge (Hoppe et al., 2002; Santoro et al., 2010). For example, runoff from winter rainstorms can influence phytoplankton dynamics (Corcoran and Shipe, 2011) which could in turn affect marine enzyme activities. Runoff may also provide terrestrial inputs of nutrients or microbial cells that could stimulate enzyme production (Ahn et al., 2005). However, we did not observe consistently elevated enzyme activities in winter or in conjunction with low salinity as we would expect if enzymes were influenced by terrestrial runoff.

We frequently observed “blooms” of enzyme activity that were not clearly related to offshore or local environmental drivers. These blooms are probably associated with the advection or growth of specific microbial groups (Martinez et al., 1996; Riemann and Steward, 2000). For example, a mesocosm study with marine microbes from the Southern California Bight found that elevated enzyme activities following a phytoplankton bloom coincided with increased abundances of the bacterial clades *Cytophaga*, *Roseobacter*, *Alteromonas*, and α-*Proteobacteria* (Riemann and Steward, 2000). *Cytophaga* in particular are known to produce enzymes and associate with particles (Delong et al., 1993). For BG and LAP, variation in enzyme activity may reflect the dynamics of microbial taxa that specialize on particle hydrolysis.

The idea that particles of marine snow are hotspots for bacterial enzyme production and hydrolysis has been established for some time (Karner and Herndl, 1992; Smith et al., 1992; Hoppe et al., 2002). However our results for NAG and particularly AP are consistent with other studies demonstrating that a substantial fraction of extracellular enzyme activity may be freely dissolved in the water column (Obayashi and Suzuki, 2008; Baltar et al., 2010). NAG is produced by a variety of organisms, and free NAG may be derived from molting crustaceans, such as copepods, rather than bacteria which tend to produce particle-bound NAG (Oosterhuis et al., 2000). The high proportion of AP we observed in the free fraction is surprising because AP is commonly located in the periplasmic space, and microbes cannot readily access the hydrolysis products of enzymes released into a well-mixed environment (Allison, 2005). One possible explanation for high AP activity in the free fraction is that filtration releases periplasmic or bound enzymes. However, we are uncertain as to why such an artifact would have a strong effect on AP compared to the other enzymes. Another possibility is that AP released after cell death has a long residence time in the free water fraction (Steen and Arnosti, 2011). This explanation is unlikely because we observe high short-term variation in activity, meaning that turnover processes must remove activity on similar timescales to enzyme production. A third explanation is that there is an advantage to AP release, such as access to substrate. There are large pools of dissolved nucleic acids in seawater, and perhaps the P in these compounds can only be accessed by free enzymes (Dell’Anno and Danovaro, 2005; Lennon, 2007).

Taken together, our results demonstrate that extracellular enzyme activities are highly variable on short temporal scales in coastal southern California. Relationships with chlorophyll and nutrients imply that at least some of this variation is related to the dynamics of phytoplankton and primary production. However, these drivers explain less than 50% of the variation in enzyme activity, suggesting that population dynamics of enzyme-producing organisms may be underlying the enzymatic changes. Although we did not measure these organisms directly, our data suggest that their populations probably vary on shorter timescales than the seasonally varying bacterial communities observed at SPOTS and the English Channel (Fuhrman et al., 2006; Gilbert et al., 2012). Furthermore, the enzymes catalyzing carbohydrate and protein degradation (BG and LAP) likely relate to bacterial populations on particles, whereas enzymes related to phosphorus release and chitin breakdown (AP and NAG) are dissolved in the water column and may have non-bacterial sources. To the extent that extracellular enzyme activities control remineralization of marine organic matter, our results imply that nutrient regeneration in coastal environments may also vary at fine temporal scales.

## Conflict of Interest Statement

The authors declare that the research was conducted in the absence of any commercial or financial relationships that could be construed as a potential conflict of interest.

## Footnotes

^1^www.sccoos.org

^2^http://oceancolor.gsfc.nasa.gov/

^3^http://oceancolor.gsfc.nasa.gov/seadas/

^4^http://www.pfel.noaa.gov/products/PFEL/modeled/indices/upwelling/NA/
